# Nanopore direct RNA sequencing reveals *N*^6^-methyladenosine and polyadenylation landscapes on long non-coding RNAs in *Arabidopsis thaliana*

**DOI:** 10.1186/s12870-024-05845-4

**Published:** 2024-11-26

**Authors:** Qiaoxia Liang, Jizhou Zhang, Hon-Ming Lam, Ting-Fung Chan

**Affiliations:** School of Life Sciences and State Key Laboratory of Agrobiotechnology, The Chinese University of Hong Kong, Shatin, Hong Kong SAR China

**Keywords:** Long non-coding RNA, *Arabidopsis thaliana*, *N*^6^-methyladenosine, Polyadenylation, Stage development

## Abstract

**Background:**

Long non-coding RNAs (lncRNAs) play important roles in various biological processes, including stage development in plants. *N*^6^-methyladenosine (m^6^A) modification and polyadenylation are noteworthy regulatory processes that impact transcript functions by modulating their abundance. However, the specific landscapes of m^6^A modification and polyadenylation on lncRNAs remain largely unexplored. The advent of nanopore direct RNA sequencing (DRS) provides unprecedented opportunities for directly detecting m^6^A modifications and estimating polyadenine (poly[A]) tail lengths on individual RNA molecules.

**Results:**

Here we utilized nanopore DRS to identify lncRNAs and map the transcriptome-wide m^6^A modification and polyadenylation landscapes in the model plant *Arabidopsis thaliana*. Leveraging the Low-abundance Aware Full-length Isoform clusTEr (LAFITE) assembly pipeline, we identified 1149 novel lncRNAs in seventeen nanopore DRS datasets from the wild-type Columbia-0. Through the precise detection of 2381 m^6^A modification sites on lncRNAs at single-base resolution, we observed that lncRNAs exhibited lower methylation levels compared to protein-coding RNAs, and m^6^A modification facilitated lncRNA abundance. Additionally, we estimated the poly(A) tail lengths of individual lncRNAs and found that poly(A) tails contributed to lncRNA stability, while their effect was not length-dependent. Furthermore, by comparing lncRNA abundance between 2-week seedlings and 5-week floral buds, we revealed the dynamic expression patterns of lncRNAs during the transition from the vegetative stage to the reproductive stage. These observations provided insights into their potential roles in specific tissues or stages in *Arabidopsis*, including regulating stage development. Moreover, by integrating information on m^6^A modification, we unveiled a positive correlation between methylation variances and differential expressions of lncRNAs during stage development.

**Conclusions:**

These findings highlighted the significance of epigenetic modification and post-transcriptional processing in shaping lncRNA expression and their functions during *Arabidopsis* stage development, contributing to the growing field of lncRNA research in plants.

**Clinical trial number:**

Not applicable.

**Supplementary Information:**

The online version contains supplementary material available at 10.1186/s12870-024-05845-4.

## Background

Long non-coding RNAs (lncRNAs) have gained increasing attention in recent decades with their active involvement in various biological processes, including stage development. In plant species like *Arabidopsis thaliana*, lncRNAs play pivotal roles in essential developmental processes such as root formation and flowering regulation [1–5]. For instance, the lncRNA *COLDAIR*, situated within the intronic region of *FLC*, is activated by the transcription factor WRKY63 during vernalization, leading to the repression of *FLC* expression and regulation of flowing time [1]. In addition, a hypervariable structural element of *COLDAIR*, complementary to the *FLC* transcription start site, exerts regulatory control over *FLC* expression and the flowering time of *Arabidopsis* [2]. Recently, the lncRNA *APOLO* has emerged as a crucial chromatin regulator, impacting various aspects of *Arabidopsis* development, including root hair elongation, shoot branching and leaf hyponasty [3, 4]. Furthermore, the intergenic lncRNA *FLAIL* suppresses flowering in *Arabidopsis* by modulating alternative splicing and promoting the expression of its flowering target *LAC8* [5]. Despite their functional importance, lncRNAs remain inadequately characterized, particularly in post-transcriptional modifications and processing.

Post-transcriptional modifications and processing of RNAs play critical roles in regulating RNA abundance, thereby influencing intricate biological processes in plants. The methylation on adenosine to form *N*^6^-methyladenosine (m^6^A) is one of the most prevalent post-transcriptional modifications in eukaryotic RNAs. In *Arabidopsis*, researchers have proposed distinct m^6^A methyltransferase systems responsible for installing m^6^A modifications on RNAs. The most well-characterized m^6^A methyltransferase complex consists of two core methyltransferases, mRNA adenosine methylase (MTA) and MTB, along with several accessory subunits including FKBP12-interacting protein 37 kDa (FIP37), VIRILIZER (VIR), and an E3 ubiquitin-protein ligase HAKAI [6–9]. Despite the methyltransferase activity of MTA was initially observed on protein-coding RNAs and named mRNA adenosine methylase, recent findings indicate that MTA also deposits m^6^A modifications on microRNA (miRNA) precursors. This process regulates the secondary structures of pri-miRNAs, ensuring appropriate levels of mature miRNAs in *Arabidopsis* [10]. This advancement hints that various RNA types might share or partially share identical m^6^A methyltransferase mechanisms. Apart from the MTA and MTB complex, FIONA1 (FIO1) also exhibits m^6^A methyltransferase properties on multiple RNA types. FIO1 is responsible for m^6^A modification on over 2000 transcripts, predominantly in the coding sequences of a subset of protein-coding RNAs [11]. This action regulates the transcript abundance of the flowering integrator *SOC1* and its upstream regulators, thereby controlling flowering time in *Arabidopsis* [11]. Additionally, FIO1 introduces m^6^A modifications on U6 small nuclear RNA [12]. Recently, a novel m^6^A methyltransferase, ATMETTL5, was discovered to be responsible for installing m^6^A methylation on a specific site of 18S ribosomal RNA (rRNA). This procedure coordinates blue light-mediated hypocotyl growth by regulating the translation of blue-light-related protein-coding RNAs in *Arabidopsis* [13]. Although the methylation mechanism on lncRNAs has yet to be elucidated, m^6^A modifications have been observed on lncRNAs in *Arabidopsis*. Notably, the lncRNA *COOLAIR* undergoes MTA-mediated m^6^A modification, influencing the dynamics of FCA nuclear condensates and promoting *FLC* chromatin silencing in *Arabidopsis* [14]. However, the predominant focus on protein-coding RNAs in m^6^A modification research has left the m^6^A landscape on lncRNAs largely unexplored in *Arabidopsis*. Despite this gap, structural similarities in the 5’ cap and 3’ polyadenylated (poly[A]) tail suggested that lncRNAs may share methyltransferase complex with protein-coding RNAs. This assumption was supported by the MTA-mediated m^6^A methylation on *COOLAIR* in *Arabidopsis*, and the fact that lncRNAs are extensively methylated by METTL3, the orthologue of MTA, in *Homo sapiens* [14, 15].

Polyadenylation, also known as poly(A) tailing, is a crucial post-transcriptional processing that governs RNA metabolism. The addition of adenosines at the 3’ end of the transcripts enhances RNA stability by interacting with poly(A)-binding proteins and shielding them from exonucleolytic degradation [16]. In *Arabidopsis*, the poly(A) tail lengths of protein-coding RNAs have been extensively characterized, and their correlation with protein-coding RNA abundance has been elucidated [17, 18]. While a substantial proportion of lncRNAs possess poly(A) tails, not all of them exhibit this characteristic. The presence of poly(A) tails globally contributes to lncRNA abundance [19]. Nevertheless, understanding of polyadenylation and its role in regulating lncRNA abundance remains limited.

Previous efforts on identifying and characterizing lncRNAs in *Arabidopsis* have mainly relied on high-throughput but short-read RNA sequencing. However, the advent of nanopore direct RNA sequencing (DRS) coupled with state-of-the-art bioinformatic tools has revolutionized research on the *Arabidopsis* transcriptome [18]. Nanopore DRS directly captures native m^6^A modifications by detecting the electrical signal fluctuations introduced by methylated adenosines. In addition, the poly(A) selection and ultralong (or even full-length) sequencing of nanopore DRS enable direct estimation of the poly(A) tail lengths of individual RNA molecules. These features of nanopore DRS provide an unprecedented opportunity to investigate the roles of m^6^A modification and polyadenylation in lncRNA expression.

In this study, we aimed to leverage nanopore DRS for a comprehensive characterization of the global m^6^A modification and polyadenylation patterns in lncRNAs, and the investigation of their contributions to lncRNA expression dynamics during stage development in *Arabidopsis*. To capture ultralong transcripts for novel lncRNA identification, we recruited seventeen nanopore DRS datasets sourced from the wild-type *Arabidopsis*, encompassing essential developmental stages: the vegetative stage and the reproductive stage. As a result, we successfully uncovered 23,458 high-confident novel transcripts and subsequently identified 1149 novel lncRNAs from the repertoire of novel transcripts. Furthermore, we precisely mapped 2381 m^6^A-modified sites on lncRNAs at single-base resolution from the raw electrical signals of nanopore DRS data and compared the m^6^A modification features between lncRNAs and protein-coding RNAs. By comparing with m^6^A-modified sites detected from various DRS datasets derived from mutants of VIR, FIP37, MTB or FIO1, we proposed the potential that there may be additional methyltransferases apart from the well-known MTA complex and FIO1. Furthermore, our analysis of polyadenylation landscape shed light on the intricate regulatory roles governed by poly(A) tails, elucidating their profound impact on lncRNA expression. Finally, we focused on 2-week seedlings and 5-week floral buds and characterized the effects of m^6^A modifications and poly(A) tails on the differential expression patterns of lncRNAs when the plants transitioned from the vegetative stage to the reproductive stage. Overall, our findings revealed the distinct characteristics of m^6^A modifications and poly(A) tails of lncRNAs in *Arabidopsis*, providing valuable insights into the intricate regulatory effects of the post-transcriptional processing on lncRNA expression dynamics during stage development.

## Methods

### Data collection

To construct a more comprehensive transcriptome for lncRNA identification in *Arabidopsis*, we curated a diverse collection of seventeen nanopore DRS datasets derived from wild-type Columbia-0 (Col-0). Among these datasets, four were generated in-house, including two 2-week seedling (stage 1.04) libraries and two 5-week floral bud (stage 6.0-6.10) libraries [20]. Additionally, we incorporated ten 2-week seedling datasets [18, 21] and three 6-day seedling (stage 1.0) datasets [11] acquired from external sources. All seventeen datasets were also utilized to call m^6^A methylome and estimate the poly(A) tail length.

To investigate the potential involvement of additional methyltransferases responsible for lncRNA methylation apart from MTA complex and FIO1, we also included four *vir-1* mutant datasets [18], three *fip37-4* mutant datasets [21], three *mtb* mutant datasets [21], and three *fio1-2* mutant datasets [11] for comparison with the m^6^A methylome of wild-type *Arabidopsis*.

To capture non-poly(A) lncRNAs and estimate the expression levels of transcripts, we obtained four 7-day seedling (stage 1.0) datasets and 47-day (stage 6.9) datasets from Illumina next-generation sequencing (NGS) [22].

Detailed information regarding the collected datasets was provided in Table S1.

### Nanopore DRS data processing and transcriptome assembly

The TAIR10 reference genome and Araport11 annotation of *Arabidopsis* were acquired from Ensembl Plants for alignment and lncRNA identification [23]. Raw electrical signals from nanopore DRS were processed in Guppy (version 6.4.8) to enable base-calling and adapter trimming. The read quality and read length were assessed to validate the library quality using NanoStat (version 1.6.0) [24]. The called sequences were aligned to the reference genome using Minimap2 (version 2.24) with a *k*-mer size of 14 (-k 14) and a maximum intron length of 500 kilobases (-G 500,000) [25]. The polyadenylation events indicating completion of the sequencing process were detected on aligned reads using Nanopolish (version 0.14.0) [26].

The transcript assembly of each library was individually constructed using the Low-abundance Aware Full-length Isoform clusTEr (LAFITE) pipeline with the default parameters and guided by the reference genome and annotation [27]. The individual transcript assemblies were further conflated into a non-redundant transcriptome. Transcripts supported by a minimum of two full-length reads were regarded as *bona fide* transcripts and retained for further analyses.

### Novel lncRNA identification and classification

Novel transcripts were identified by a comparison with the reference annotation using GffCompare (version 0.12.1) [28]. Transcripts labelled with class code “=” were excluded as they represented annotated transcripts. The coding probabilities of the remaining transcripts were predicted using CPC2 (version 1.0.1) and CPAT (version 3.0.4) with default parameters [29, 30]. For CPAT, a training dataset consisting of randomly selected coding sequences (CDS) and non-coding sequences from the untranslated region (UTR) of *Arabidopsis* was prepared to determine the coding probability cutoff for classifying coding and non-coding transcripts. The optimum cutoff was 0.325 and was determined using a two-graph receiver operating characteristic (TG-ROC) curve in the ROCR package [31]. The performance of sensitivity and specificity under this cutoff was 0.957. Novel transcripts exceeding 200 nucleotides (nt) in length with a coding probability below 0.325 reported by CPAT and marked as “non-coding” by CPC2 were compiled as potential novel lncRNAs. Any potential novel lncRNAs with splice junctions matching those of protein-coding RNAs were excluded from subsequent analyses.

The novel lncRNAs along with annotated lncRNAs obtained from Araport11 annotation were then categorized into antisense lncRNAs, intergenic lncRNAs, intronic lncRNAs, and overlapping lncRNAs using GffCompare (version 0.12.1) [28]. Specifically, lncRNAs with class code “s” or “x” were classified as antisense lncRNAs; those with class code “p” or “u” were classified as intergenic lncRNAs; those with class code “i” were classified as intronic lncRNAs; and those with class code “y” or “o” were classified as overlapping lncRNAs.

### Expression estimation and differential expression analysis

To estimate the expression levels of transcripts, DRS reads were aligned to the reconstructed transcriptome containing non-redundant novel and annotated transcripts using Minimap2 (version 2.24) [25]. The minimum secondary-to-primary score ratio was set at 0.8 (-p 0.8), with a retention of up to 10 secondary alignments (-N 10). Subsequently, raw counts of individual transcripts in each library were estimated using Salmon (version 1.10.0) in the alignment-based mode for Oxford nanopore long reads (--ont) [32]. These estimated counts were then normalized into counts per million (CPM). A transcript with a CPM exceeding 0.1 in a sample was considered moderately expressed in the sample.

The differential expression (*d*) of each transcript was calculated using the following formula:

$$\:d={\text{log}}_{2}\frac{\frac{\sum\:_{i=1}^{n}\left({x}_{i}\right)}{n}+0.1}{\frac{\sum\:_{j=1}^{m}\left({y}_{j}\right)}{m}+0.1}$$

where *n* is the total number of floral bud samples, *x*_*i*_ is the CPM of the transcript in a given floral bud sample, *m* is the total number of seedling samples, and *y*_*i*_ is the CPM of the transcript in a given seedling sample. A constant of 0.1 was set as a pseudo-CPM. The resulting *d* values indicated the expression differences between transcripts in the vegetative stage (2-week seedling) and the reproductive stage (5-week floral bud). Transcripts with *d* exceeding 0.7 were regarded as upregulated in the floral bud, while those with *d* below -0.7 were considered as downregulated in the floral bud.

### Construction of m^6^A methylome and metagene profiles

To transcriptome-wide detect m^6^A modifications and construct the m^6^A methylome for *Arabidopsis*, DRS reads from each library were aligned to the reconstructed transcriptome using Minimap2 (version 2.24), with exclusion of any multi-aligned reads (--secondary = no) [25]. The methylated sites on each transcript were detected by analysing the raw electrical signal fluctuations in conjunction with the uniquely aligned reads using m6Anet with a pre-built *vir-1* complemented (VIRC) mutant model of *Arabidopsis* [33]. Potential methylation sites with fewer than five supporting reads were excluded to ensure the reliability of m^6^A detection (--min_segment_count 5).

To exhibit metagene profiles of m^6^A-modified sites on lncRNAs and protein-coding RNAs, we converted the transcript coordinates of m^6^A sites into their relative positions on the transcripts. For lncRNAs, the m^6^A sites were normalized based on their relative positions on the entire transcript. For protein-coding RNAs, the transcripts were segmented and normalized into relative sizes with a ratio of 5’ UTR: CDS: 3’ UTR = 2: 5: 3. Subsequently, the transcript coordinates of m^6^A sites on protein-coding RNAs were accordingly converted to their relative positions within the regions.

The transcript-level m^6^A modification ratios (*M*) were calculated as follows:

$$\:M=\frac{\sum\:_{i=1}^{n}({m}_{i}\times\:{r}_{i})}{\sum\:_{i=1}^{n}{r}_{i}}$$

where *m*_*i*_ is the modification ratio of a detected m^6^A site on a specific transcript, *r*_*i*_ is the number of supporting reads for the detected locus, and *n* is the total number of all detected m^6^A sites on the transcript.

The differential modification ratio of a transcript was defined as the variance between the average transcript-level methylation ratios in the 5-week floral bud libraries and those in the 2-week seedling libraries. A positive variance represents an increasing m^6^A modification level of the transcript during stage development, while a negative variance indicates a decreasing methylation level of the transcript during stage development.

### Illumina NGS data processing and expression estimation

The raw sequencing reads were pre-processed using Cutadapt (version 4.1) to trim adapters and low-quality reads [34]. The ribosomal RNA (rRNA) and transfer RNA (tRNA) sequences of *Arabidopsis* were obtained from Ensembl Plants. Subsequently, rRNA and tRNA sequences were removed from the trimmed reads using Bowtie2 (version 2.4.2) [35]. The estimated raw counts and normalized transcripts per million (TPM) of individual transcripts in each library were calculated using Kallisto (version 0.46.1) with the pseudoalignment strategy [36].

### Polyadenylation identification and poly(A) tail length estimation

To characterize the polyadenylation diversity in lncRNAs, we categorized them into poly(A) lncRNAs and non-poly(A) lncRNAs. Given that nanopore DRS libraries employed poly(A) selection to enrich target transcripts, whereas Illumina NGS libraries utilized rRNA depletion, we designated a lncRNA as a non-poly(A) lncRNA if it was detected in any Illumina NGS dataset with TPM > 0.1 in at least two samples, yet no read passing quality control was found in any nanopore DRS libraries. Conversely, the remaining lncRNAs detected in nanopore DRS data and meeting the criteria of TPM > 0.1 in at least two Illumina NGS samples were classified as poly(A) lncRNAs.

To estimate the poly(A) tail length of each transcript, we calculated the poly(A) tail lengths of individual DRS reads uniquely mapped to the reconstructed transcriptome using Nanopolish (version 0.14.0) [26]. Subsequently, the poly(A) tail length of each transcript was calculated by averaging the poly(A) tail lengths of all DRS reads passing quality control and aligned to that transcript.

## Results

### Identification and characterization of lncRNAs in *Arabidopsis*

To identify novel lncRNAs in *Arabidopsis*, we retrieved a substantial collection of nanopore DRS datasets from different developmental stages of wild-type Col-0. In total, we captured 25.60 million high-quality reads. The average read length of each library ranged from 767 to 1079 nt, indicating the ability of nanopore DRS in capturing ultralong RNA molecules. Their primary alignment rates surpassed 90%, except for two libraries incorporating cap-dependent 5’ adapter ligation. Ultimately, we acquired 24.09 million uniquely mapped reads for transcriptome assembly and novel transcript identification.

**Fig. 1:**
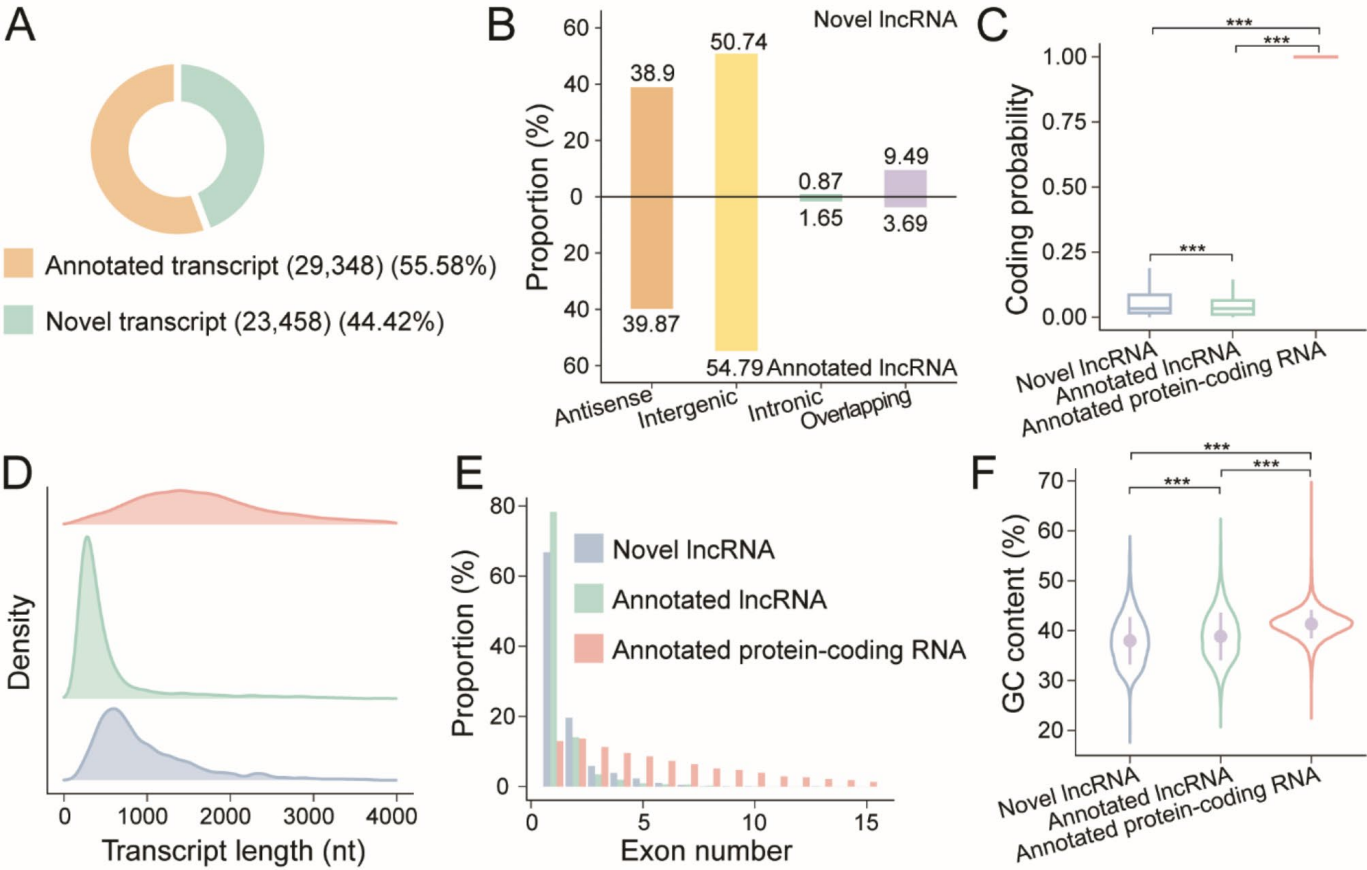
Identification and characterization of lncRNAs in *Arabidopsis*. (**A**) Pie chart showing the numbers and proportions of annotated transcripts from Araport11 (orange) and novel transcripts from nanopore DRS transcriptome assembly (green). (**B**) Bar chart exhibiting the proportions of lncRNA categories in novel lncRNAs (from nanopore DRS assembly) and annotated lncRNAs (from Araport11) according to their genomic positions relative to their neighbouring protein-coding RNAs. (**C**) Box plot comparing the coding probabilities among novel lncRNAs (blue), annotated lncRNAs (green) and annotated protein-coding RNAs (red). The coding probabilities of lncRNAs were close to 0 and were significantly inferior to those of protein-coding RNAs, which were close to 1 (Mann-Whitney *U* test; ***, *p* < 0.001). (**D**) Density plot presenting the distributions of transcript lengths of novel lncRNAs (blue), annotated lncRNAs (green) and annotated protein-coding RNAs (red). LncRNAs tended to be shorter than protein-coding RNAs. (**E**) Histogram comparing the exon numbers among novel lncRNAs (blue), annotated lncRNAs (green) and annotated protein-coding RNAs (red). The majority of lncRNAs had fewer than two exons, while protein-coding RNAs had a broader range of exon numbers. (**F**) Violin plot comparing the GC contents among novel lncRNAs (blue), annotated lncRNAs (green) and annotated protein-coding RNAs (red). The GC contents of lncRNAs were significantly lower than those of protein-coding RNAs (Mann-Whitney *U* test; ***, *p* < 0.001)

The uniquely aligned reads were assembled using the LAFITE pipeline to construct a comprehensive transcriptome of *Arabidopsis*. The assemblies yielded 52,806 non-redundant transcripts from 23,821 genomic loci. Among the assembled transcripts, 29,348 were previously annotated in the Araport11 reference annotation, accounting for 55.58% of the reconstructed transcriptome (Fig. 1A). The moderate proportion of annotated transcripts highlighted the high quality and fidelity of the reconstructed transcriptome, and thus its suitability for lncRNA identification.

Nanopore DRS and LAFITE assembly provided 23,458 novel transcripts for lncRNA prediction (Fig. 1A). We first predicted the coding probabilities of novel transcripts and compiled a list of potential lncRNAs with inferior coding probabilities. We next filtered out transcripts with splice junctions matching any splice junctions of protein-coding transcripts, as they could be novel isoforms of protein-coding transcripts. Together with 3879 annotated lncRNAs in Araport11, the remaining 1149 novel lncRNAs were classified into different categories according to their positions relative to their neighbouring protein-coding RNAs. This classification revealed that the novel lncRNAs comprised 447 (38.90%) antisense lncRNAs, 583 (50.74%) intergenic lncRNAs, 10 (0.87%) intronic lncRNAs, and 109 (9.49%) overlapping lncRNAs (Fig. 1B; Table S2). Similarly, we found that the annotated lncRNAs comprised 1523 (39.87%) antisense lncRNAs, 2093 (54.79%) intergenic lncRNAs, 63 (1.65%) intronic lncRNAs, and 141 (3.69%) overlapping lncRNAs (Fig. 1B; Table S2).

To gain further insight into lncRNAs, we compared their sequence properties to those of protein-coding RNAs. The results revealed distinct features of lncRNAs (Fig. C-F). First, lncRNAs showed relatively poor coding probabilities (Fig. 1C), highlighting their non-coding nature. Furthermore, lncRNAs were generally shorter (Fig. 1D), had fewer exons (Fig. 1E), and had significantly lower GC contents (Fig. 1F) than protein-coding RNAs. Specifically, the average lengths of novel lncRNAs and annotated lncRNAs were 983 and 602 nt respectively. In contrast, protein-coding RNAs had an average length of 1788 nt, significantly surpassing that of lncRNAs (Mann-Whitney *U* test, *p* < 0.001; Fig. 1D). Meanwhile, lncRNAs tended to have fewer exons, with a substantial proportion (86.33% of novel lncRNAs and 92.37% of annotated lncRNAs) consisting of only one or two exons. By contrast, protein-coding RNAs exhibited a broader range of exon numbers (Fig. 1E). This observation regarding the exon numbers of transcripts implied that lncRNAs underwent fewer splicing events than protein-coding RNAs. Lastly, lncRNAs demonstrated significantly lower GC contents than protein-coding RNAs, with median GC contents of 37.65% for novel lncRNAs and 38.73% for annotated lncRNAs. In comparison, protein-coding RNAs displayed a median GC content of 41.36%, significantly higher than that of lncRNAs (Mann-Whitney *U* test, *p* < 0.001; Fig. 1F).

Additionally, we delineated the characteristics of lncRNAs across various categories. While the coding probabilities of lncRNAs generally appeared poor and their GC contents were relatively low, we observed that overlapping lncRNAs and antisense lncRNAs exhibited comparatively higher coding probabilities and GC contents compared to intergenic lncRNAs and intronic lncRNAs (Fig. S1A & B). This discrepancy could be attributed to the genomic locations where overlapping lncRNAs intersected with protein-coding RNAs, and where antisense lncRNAs resided on the opposite strand relative to protein-coding RNAs. In contrast, intergenic lncRNAs and intronic lncRNAs showed distinct coding probabilities and GC contents, setting them apart from the exon regions of protein-coding RNAs. Notably, although the length distributions of various lncRNA categories appeared similar, the average lengths of antisense lncRNAs (595 nt) and overlapping lncRNAs (583 nt) exceeded those of intergenic lncRNAs (348 nt) and intronic lncRNAs (272 nt) (Fig. S1C). The notably shorter average length of intronic lncRNAs could be explained by the limited intronic regions between the splice junctions of protein-coding RNAs. Furthermore, the distributions of transcript lengths also explained the exon number distributions among lncRNA categories (Fig. S1D). All intronic lncRNAs with the shortest average length consisted of a single exon. By contrast, a significant proportion of antisense lncRNAs (30.46%) and overlapping lncRNAs (35.60%), which exhibited longer average lengths, contained multiple exons. Only 18.24% of intergenic lncRNAs underwent splicing.

The distinctive features of lncRNAs in comparison to protein-coding RNAs aligned well with findings reported in prior studies on lncRNAs in *Arabidopsis* and other plant species that have primarily relied on short-read NGS techniques [37–39]. This alignment confirmed the robustness of our lncRNA identification approach based on nanopore DRS and LAFITE assembly. Moreover, our study provided a more advanced characterization among various lncRNA categories, revealing the nuanced features and distinctions present within these varied categories of lncRNAs. In summary, our efforts in lncRNA identification and characterization in *Arabidopsis* laid a foundation for exploring the epigenetic modification and post-transcriptional processing features of lncRNAs.

### The *N*^6^-methyladenosine landscape of *Arabidopsis* lncRNAs

**Fig. 2:**
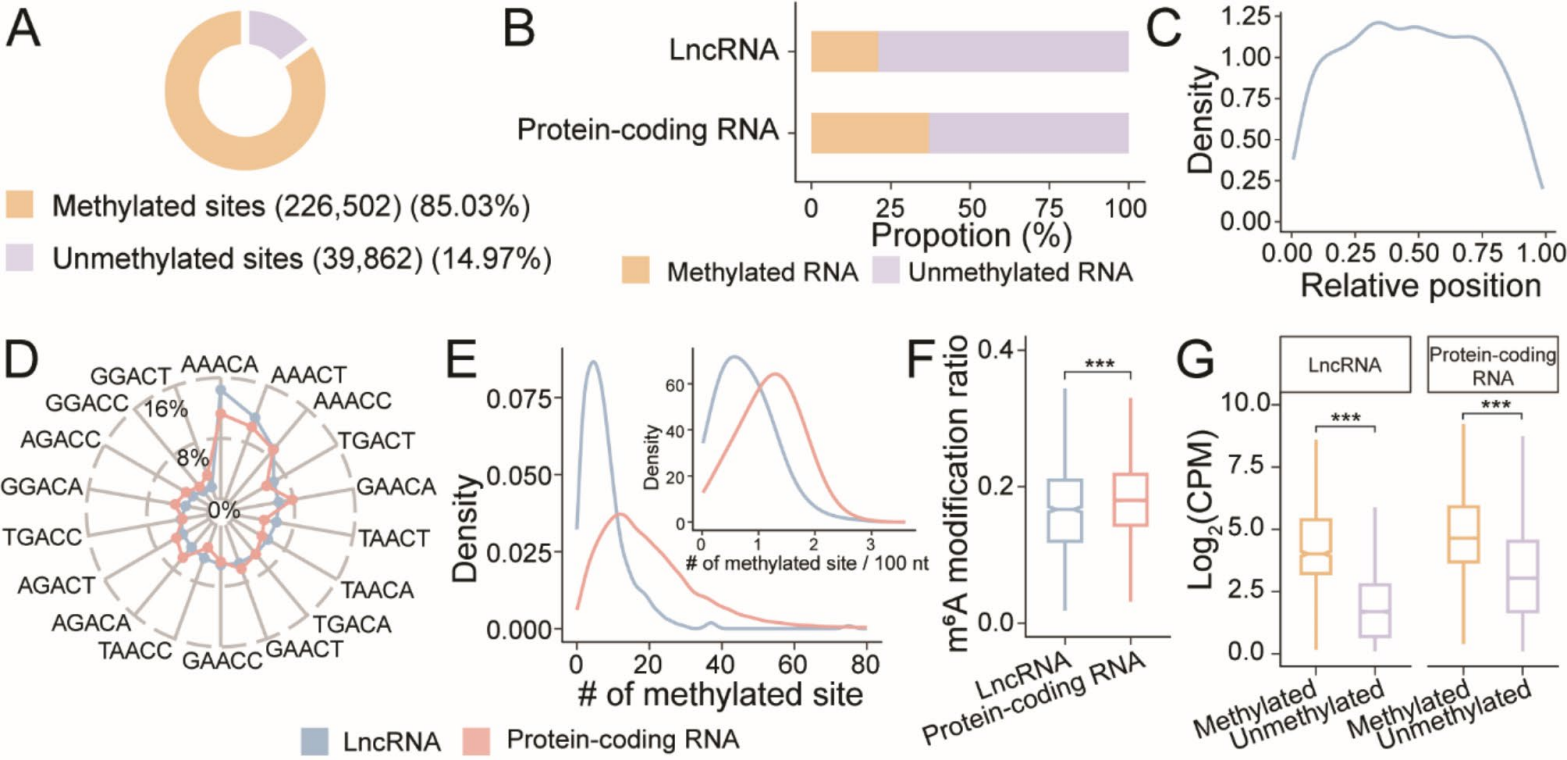
The transcriptome-wide m^6^A landscape of *Arabidopsis* lncRNAs. (**A**) Pie chart showing the numbers and proportions of methylated sites and unmethylated sites embedded in the canonical DRACH motifs (where D = A/T/G, R = A/G, and H = A/T/C). (**B**) Bar chart demonstrating the proportions of methylated RNAs and unmethylated RNAs in different types of transcripts. LncRNAs had a lower proportion of methylated RNAs than protein-coding RNAs. (**C**) Metagene profile depicting the relative positions of m^6^A-modified sites on lncRNAs. (**D**) Radar plot exhibiting the 5-mer motif usage of methylated sites on lncRNAs (blue) and protein-coding RNAs (red). The m^6^A-modified sites were preferentially located in the AAACH motifs on both types of transcripts. (**E**) Density plots presenting the distributions of numbers and densities of m^6^A-modified sites on lncRNAs (blue) and protein-coding RNAs (red). LncRNAs tended to have significantly fewer methylated sites and a lower methylated density than protein-coding RNAs. (**F**) Box plot comparing the transcript-level modification ratios between lncRNAs (blue) and protein-coding RNAs (red). LncRNAs were significantly less methylated than protein-coding RNAs (Mann-Whitney *U* test; ***, *p* < 0.001). (**G**) Box plots showing the abundance of methylated RNAs (orange) versus unmethylated RNAs (purple) in lncRNAs and protein-coding RNAs. The expression levels of methylated RNAs were significantly higher than those of unmethylated RNAs in both types of transcripts (Mann-Whitney *U* test; ***, *p* < 0.001)

Previous studies have highlighted the role of m^6^A modification in regulating lncRNA expression and influencing their functions in human [40]. Nevertheless, the landscape of m^6^A modification on lncRNAs in plants has not been extensively investigated. Here we employed nanopore DRS to capture native RNA molecules, preserving nucleotide modification signals on individual transcripts. This approach allowed us to directly detect m^6^A modifications across the transcriptome and achieve single-base resolution. Our efforts unveiled a total of 266,364 potential m^6^A sites embedded in the canonical DRACH 5-mer motif (where D = A/T/G, R = A/G, and H = A/T/C) within the reconstructed transcriptome of *Arabidopsis*. Among these potential m^6^A sites, 226,502 (85.03%) were methylated sites, with a modification ratio exceeding 0, as reported by m6Anet. These methylated sites were found on 12,165 transcripts across seventeen wild-type nanopore DRS libraries (Fig. 2A).

Following the detection of methylated sites, we annotated them to different transcript categories. A transcript harbouring at least one m^6^A-modified site was classified as a methylated transcript. Our analysis revealed 200,854 methylated sites on 9498 protein-coding RNAs, accounting for 37.03% of 25,650 moderately expressed protein-coding RNAs with a CPM exceeding 0.1 in any library (Fig. 2B). In contrast, 2381 methylated sites were found on 293 (21.12%) of 1387 moderately expressed lncRNAs, indicating a lower prevalence compared to protein-coding RNAs (Fig. 2B; Table S3). Furthermore, the distribution of m^6^A sites along lncRNAs showed an approximately uniform pattern, but notably distancing from the transcription start site (TSS) and the transcription termination site (TTS) (Fig. 2C). Simultaneously, we assigned the m^6^A-modified sites along various regions of the gene body on protein-coding RNAs. Irrespective of modification ratios, we observed a distinct enrichment of methylated sites on the coding sequence (CDS) region, particularly in proximity to the stop codon (Fig. S2A). This observation aligned with prior studies on m^6^A modification in *Arabidopsis* and *Oryza sativa* [11, 41]. Nevertheless, upon filtering for highly methylated sites with modification ratios exceeding 0.4, the enrichment shifted towards the 3’ untranslated region (UTR) (Fig. S2B), consistent with previous research in *Arabidopsis* using an immunoprecipitation approach coupled with NGS called m^6^A-seq and nanopore DRS strategy, and a recent study in *Arabidopsis* and *Oryza sativa* employing m^6^A-SAC-seq [21, 42, 43]. This shift observed in the metagene profiles highlighted the superiority of nanopore DRS in directly detecting methylated sites with relatively low modification ratios that may have been overlooked by the traditional NGS-based approach.

Subsequently, the DRACH motif usage, m^6^A-modified site counts, and transcript-level modifications of methylated lncRNAs were compared to those of methylated protein-coding RNAs (Fig. 2D-F). We observed similarities in the canonical DRACH motif preferences between lncRNAs and protein-coding RNAs (Fig. 2D). Over 35% of the methylated sites on lncRNAs were located within the AAACH motif, consistent with previous m^6^A motif enrichment studies utilizing m^6^A-seq [42]. Using nanopore DRS, we precisely located the m^6^A-modified sites on individual transcripts and quantified the modification ratio for each locus. Notably, the distribution of m^6^A sites revealed that lncRNAs generally contained fewer m^6^A sites, with a median count of six m^6^A sites, in contrast to seventeen sites observed on protein-coding RNAs (Fig. 2E). Upon normalization to transcript length, lncRNAs displayed a lower density of methylated sites compared to protein-coding RNAs, with a median density of 0.70 methylated sites per 100 nt for lncRNAs, contrasting with 1.22 methylated sites per 100 nt for protein-coding RNAs (Fig. 2E). These observations were consistent with the length distribution disparities previously described between lncRNAs and protein-coding RNAs. Moreover, we calculated the transcript-level modification ratios for all potential m^6^A sites, regardless of their modification status. Our analysis suggested that lncRNAs, with a median modification ratio of 0.16, were significantly less methylated compared to protein-coding RNAs, which displayed a median modification ratio of 0.18 (Mann-Whitney *U* test, *p* < 0.001; Fig. 2F). Finally, a comparison of the abundances of methylated and unmethylated transcripts revealed that the expression levels of methylated transcripts were significantly higher than those of unmethylated ones (Mann-Whitney *U* test, *p* < 0.001; Fig. 2G). This outcome suggested a positive regulatory role of m^6^A modification on transcript abundance.

While m^6^A modifications on lncRNAs are prevalent, the specific mechanism of m^6^A deposition on lncRNAs in *Arabidopsis* remains uncharacterized. Here we endeavoured to investigate whether the well-known mechanisms contribute to m^6^A modifications on lncRNAs in *Arabidopsis*. To achieve this, we introduced diverse nanopore DRS datasets from mutants of MTA complex subunits (VIR, FIP37, or MTB) and FIO1. The comparisons of m^6^A-modified sites on lncRNAs in wild-type samples with those in various mutant samples revealed the complexity of the m^6^A modification mechanism involving these methyltransferases or subunits (Fig. S3A & B. Among all identified m^6^A sites on lncRNAs, 654 were undetectable in any mutant samples (Fig. S3A). Additionally, 308 methylated sites were detected in wild-type and all mutant samples, indicating potential m^6^A modification mechanisms independent of the MTA complex and FIO1 on these positions (Fig. S3A). The presence of m^6^A sites in wild-type samples but their absence in mutant samples implied the potential m^6^A modification mechanisms affected by the mutated methyltransferase or subunit (Fig. S3B). Interestingly, the mutation of VIR appeared to introduce 607 novel m^6^A sites (Fig. S3B). Despite novel methylated sites were also observed in mutants of FIP, MTB and FIO1, the abundance in the VIR mutant stood out prominently (Fig. S3B). This observation hinted at potential crosstalk among VIR-mediated m^6^A modifications.

### The polyadenylation characteristics of *Arabidopsis* lncRNAs

**Fig. 3:**
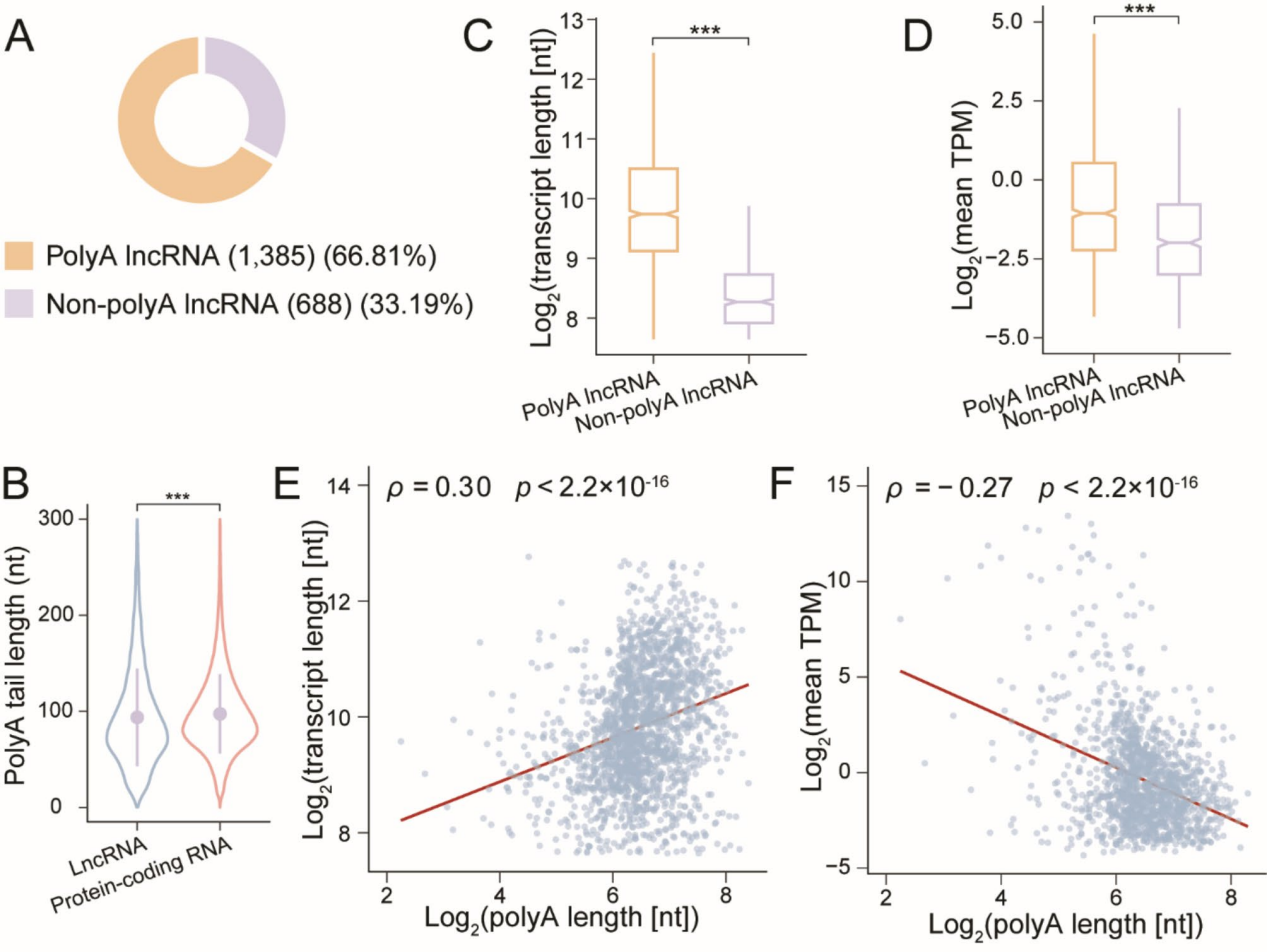
The polyadenylation landscape of *Arabidopsis* lncRNAs. (**A**) Pie chart showing the numbers and proportions of poly(A) lncRNAs and non-poly(A) lncRNAs. (**B**) Violin plot exhibiting the poly(A) tail lengths of lncRNAs (blue) and of protein-coding RNAs (red). The poly(A) tails of lncRNAs were slightly shorter than those of protein-coding RNAs (Mann-Whitney *U* test; ***, *p* < 0.001). (**C**) Box plot comparing the transcript lengths of poly(A) lncRNAs (orange) to those of non-poly(A) lncRNAs (purple). Poly(A) lncRNAs possessed significantly longer transcripts than non-poly(A) lncRNAs (Mann-Whitney *U* test; ***, *p* < 0.001). (**D**) Box plot comparing the expression levels between poly(A) lncRNAs (orange) and non-poly(A) lncRNAs (purple). Poly(A) lncRNAs were significantly more abundant than non-poly(A) lncRNAs (Mann-Whitney *U* test; ***, *p* < 0.001). (**E**) Scatter plot with linear regression demonstrating a moderately positive correlation between poly(A) tail lengths and transcript lengths of poly(A) lncRNAs (Spearman’s *ρ* = 0.30, *p* < 0.001). (**F**) Scatter plot with linear regression presenting a slightly negative correlation between poly(A) tail lengths and expression levels of poly(A) lncRNAs (Spearman’s *ρ* = -0.27, *p* < 0.001)

Nanopore DRS allowed for the direct measurement of poly(A) tail lengths on individual RNA molecules, including those of lncRNAs. However, not all lncRNAs possess a poly(A) tail. To characterize the diversity of polyadenylation among lncRNAs, we recruited a collection of Illumina NGS datasets comprising four seedling samples and four flower samples from *Arabidopsis*. These NGS datasets were prepared using a ribosomal RNA depletion strategy, therefore retaining the non-polyadenylated (non-poly[A]) lncRNAs in the libraries, which were excluded from the poly(A)-selected DRS libraries. By comparing the NGS and DRS data, we uncovered 1385 poly(A) lncRNAs, accounting for 66.81% of all the lncRNAs (Fig. 3A). In contrast, 688 non-poly(A) lncRNAs were identified, accounting for 33.19% of the lncRNAs (Fig. 3A). In summary, more than one third of lncRNAs escaped polyadenylation, indicating distinctive characteristics compared to protein-coding RNAs. To further compare the polyadenylation events between lncRNAs and protein-coding RNAs, we estimated the poly(A) tail length of individual reads and calculated the average length for each transcript. Generally, poly(A) tails of lncRNAs were slightly shorter than those of protein-coding RNAs (Fig. 3B). The median poly(A) tail length was 83.89 nt for lncRNAs and 91.09 nt for protein-coding RNAs. This discrepancy could be one of the explanations for the lower expression levels of lncRNAs compared to protein-coding RNAs, as the poly(A) tail plays a role in RNA stabilization by shielding it from exonuclease degradation [44]. Subsequently, we compared the poly(A) tail length across different categories of lncRNAs and found that intronic lncRNAs exhibited the shortest poly(A) tail length, with overlapping lncRNAs, intergenic lncRNAs, and antisense lncRNAs following in order (Fig. S1E).

Prior studies on lncRNAs in *Arabidopsis* have highlighted differences in transcript lengths and expression levels between poly(A) lncRNAs and non-poly(A) lncRNAs [19, 45]. In this study, we delved into investigating potential correlations between poly(A) tail lengths and transcript lengths, as well as between poly(A) tail lengths and lncRNA expression levels. Consistent with existing research, we observed that poly(A) lncRNAs exhibited significantly greater lengths and generally higher expression levels than non-poly(A) lncRNAs (Mann-Whitney *U* test, *p* < 0.001; Fig. 3C & D) [19, 45]. Specifically, the median length of poly(A) lncRNAs was 856 nt, whereas that of non-poly(A) lncRNAs was 309 nt (Fig. 3C). In addition, the average of mean TPM of poly(A) lncRNAs across all NGS samples was 58.19, compared to 4.14 for non-poly(A) lncRNAs (Fig. 3D). These observations aligned with the well-characterized role of poly(A) tails in stabilizing transcripts [44].

Subsequently, we focused on the correlations between transcript lengths or expression levels and poly(A) tail lengths of individual lncRNAs. As expected, there was a moderate positive correlation between the transcript lengths of individual poly(A) lncRNAs and their poly(A) tail lengths (Spearman’s *ρ* = 0.30, *p* < 0.001) (Fig. 3E). However, the TPMs of poly(A) lncRNAs showed a slight negative correlation with poly(A) tail lengths (Spearman’s *ρ* = -0.27, *p* < 0.001) (Fig. 3F). This observation was supported by prior research on protein-coding RNAs in *Arabidopsis* and *Caenorhabditis elegans*, indicating that highly expressed poly(A) transcripts tended to have shorter poly(A) tails [18, 46]. Although poly(A) tails enhanced transcript stability, our results suggested that this stabilization was not dependent on the length of the poly(A) tail. Therefore, lncRNAs with longer poly(A) tails may exhibit slightly lower expression levels compared to those with shorter poly(A) tails.

### Differential expressions of lncRNAs during stage development in *Arabidopsis*

**Fig. 4:**
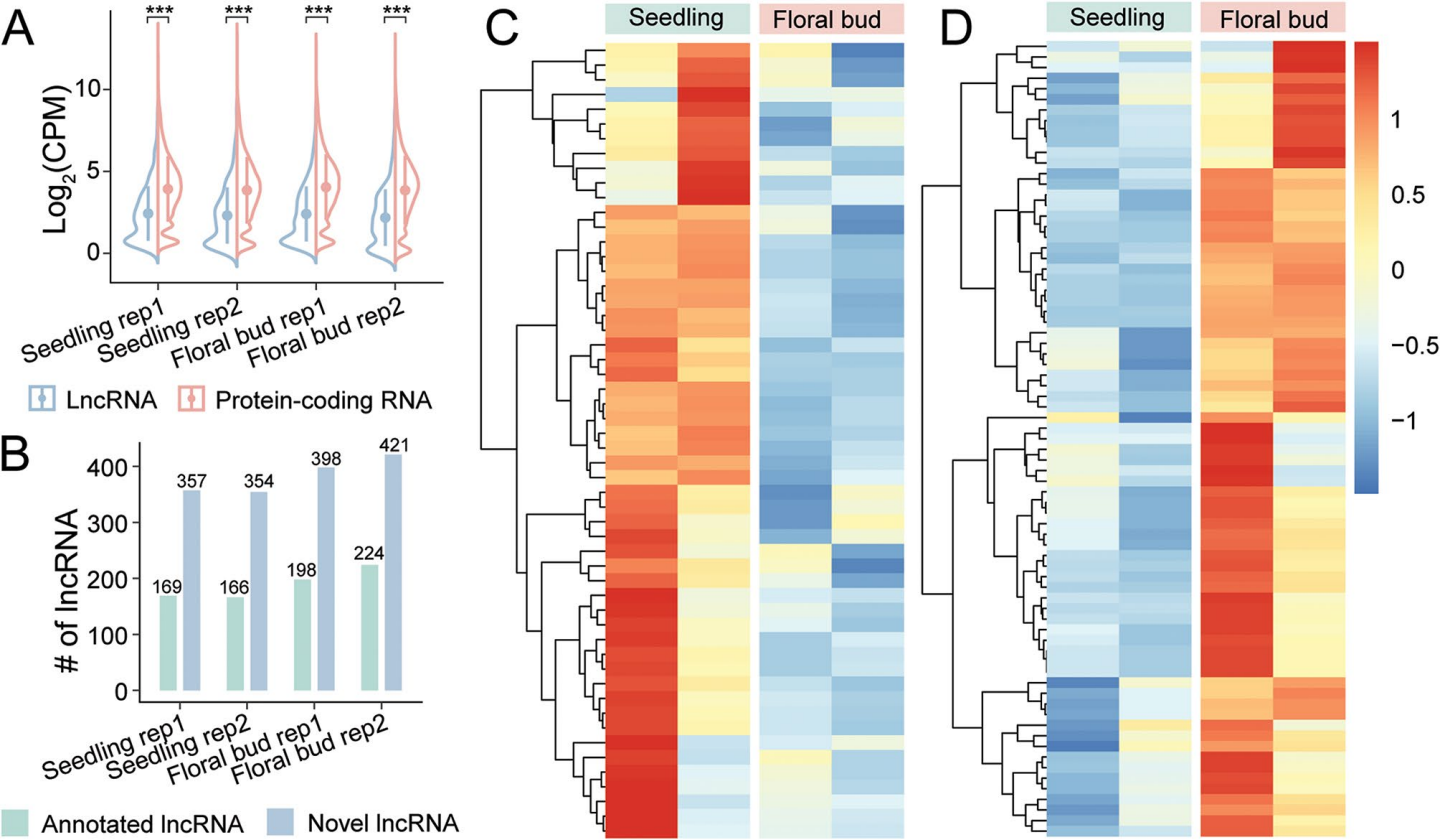
Expression dynamics of lncRNAs during stage development in *Arabidopsis*. (**A**) Violin plot comparing the expression levels between lncRNAs (blue) and protein-coding RNAs (red) at the 2-week seedling stage (vegetative stage) and the 5-week floral bud stage (reproductive stage). The expression levels of lncRNAs were significantly lower than those of protein-coding RNAs in each library (Mann-Whitney *U* test; ***, *p* < 0.001). (**B**) Bar chart showing the numbers of expressed lncRNAs in two 2-week seedling libraries and two 5-week floral bud libraries. (**C** & **D**) Heatmaps visualizing the expression levels (*z*-score-normalized CPM) of lncRNAs that were downregulated (**C**) and upregulated (**D**) during the transition from the seedling stage to the floral bud stage

In contrast to animal systems where the germ line is established in early embryogenesis, the germ line in plants forms after the successful transition from the vegetative stage to the reproductive stage. Hence, stage development is essential for plant reproduction. To investigate the involvement of lncRNAs in stage development, we estimated the expression levels of lncRNAs in our two 2-week seedling (vegetative stage) libraries and two 5-week floral bud (reproductive stage) libraries. In all four datasets, the expression levels of lncRNAs were significantly lower than those of protein-coding RNAs (Fig. 4A). This observation aligned with prior findings based on NGS data of *Arabidopsis*, affirming the accuracy of DRS quantification [39]. Thus, the precise quantification of long-read transcripts facilitated the downstream characterization of lncRNA expression patterns during stage development.

We defined a cutoff of 0.1 CPM as the threshold for moderately expressed transcripts. As a result, we detected 226 annotated lncRNAs and 451 novel lncRNAs in the seedling samples, along with 289 annotated lncRNAs and 513 novel lncRNAs in the floral bud samples (Fig. 4B; Table S4). Among these lncRNAs, 484 were expressed in both the vegetative stage and the reproductive stage. By contrast, 193 were specifically expressed in the vegetative stage, while 318 were specifically expressed in the reproductive stage. Out of the 484 lncRNAs expressed in both stages, 257 were present in all four samples. These lncRNAs were subjected to differential expression calculations, which revealed that 54 lncRNAs were downregulated during stage development and 75 lncRNAs were upregulated (Fig. 4C & D; Table S4). In summary, our identification of stage-specific lncRNAs and differentially expressed lncRNAs unveiled a subset of lncRNAs that potentially participated in the transition from the vegetative stage to the reproductive stage in *Arabidopsis*.

### The involvement of *N*^6^-methyladenosine and polyadenylation in regulating lncRNA expression during stage development

**Fig. 5:**
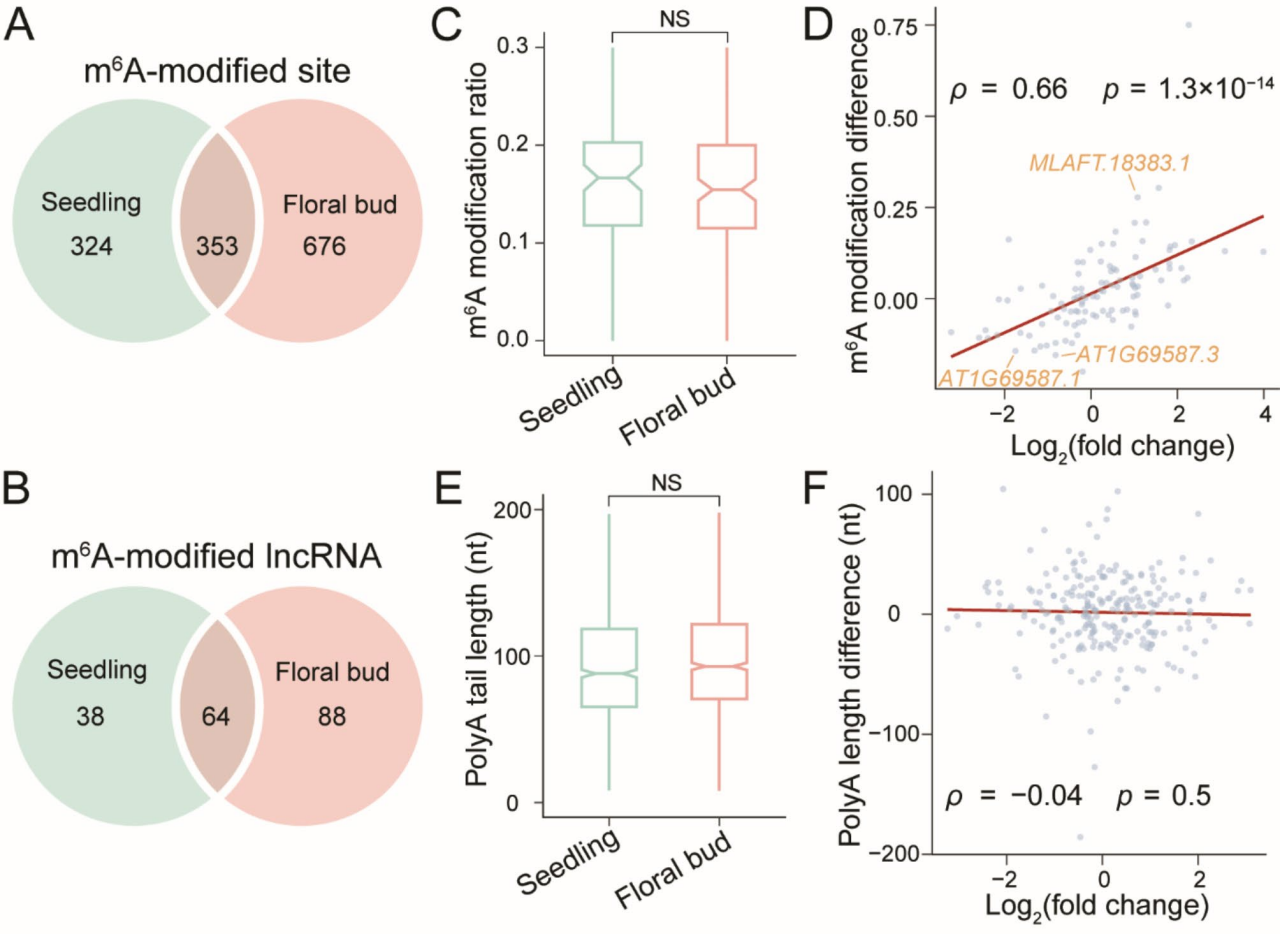
The involvement of m^6^A modification and polyadenylation in regulating lncRNA expression during stage development. (**A** & **B**) Venn diagrams showing the numbers of seedling-specific, floral-bud-specific, and shared methylated sites (**A**) and methylated lncRNAs (**B**). (**C**) Box plot exhibiting a slight decrease in global m^6^A modification ratios of lncRNAs from 2-week seedlings (vegetative stage, green) to 5-week floral buds (reproductive stage, red) (Mann-Whitney *U* test; NS, *p* > 0.1). (**D**) Scatter plot with linear regression demonstrating a significant positive correlation between differential expression fold-changes and differences in m^6^A modification ratios of lncRNAs during stage development (Spearman’s *ρ* = 0.66, *p* < 0.001). (**E**) Box plot showing a slight elongation of poly(A) tails of lncRNAs from 2-week seedlings (green) to 5-week floral buds (red) (Mann-Whitney *U* test; NS, *p* > 0.1). (**F**) Scatter plot with linear regression displaying no discernible correlation between differential expression fold-changes and differences in poly(A) tail lengths of lncRNAs during stage development (Spearman’s *ρ* = -0.04, *p* > 0.1)

To further investigate the involvement of m^6^A modification in regulating the dynamic changes in lncRNA expression during stage development, we focused on the methylated lncRNAs in 2-week seedlings and 5-week floral buds. A total of 353 distinct m^6^A-modified sites were captured on lncRNAs in both 2-week seedling libraries and 5-week floral bud libraries, accounting for 26.09% of the overall methylated sites detected on lncRNAs in four in-house samples (Fig. 5A; Table S3). By contrast, we identified 324 seedling-specific methylated sites and 676 floral-bud-specific methylated sites on lncRNAs (Fig. 5A; Table S3). At the transcript level, there were 64 methylated lncRNAs in these four libraries, with 35 methylated lncRNAs exclusively captured in the seedling stage, and 88 exclusively identified in the floral bud stage (Fig. 5B; Table S3).

During stage development, the m^6^A modification ratios exhibited a slight decline, decreasing from a median of 0.17 in 2-week seedlings to a median of 0.16 in 5-week floral buds (Fig. 5C). Nevertheless, from the vegetative stage to the reproductive stage, we observed a significant and positive correlation between the expression changes and methylation variances of 105 methylated lncRNAs that were moderately expressed in four samples (Spearman’s *ρ* = 0.66, *p* < 0.001) (Fig. 5D). Based on this observation, we inferred a general positive contribution of m^6^A modification to lncRNA expression. Hence, we focused on the differentially expressed lncRNAs with consistent methylation trends and investigated the potential functions of these lncRNAs under m^6^A regulation.

It has been noted that lncRNAs are potential of biological importance to their neighbouring protein-coding RNAs via a *cis*-regulatory mechanism in *Arabidopsis* and other plant species [47, 48]. Therefore, we endeavoured to associate the differentially expressed lncRNAs with their neighbouring protein-coding genes to elucidate their functional implications (Table S5). Interestingly, several cases were highlighted for their involvement in important signalling pathways in *Arabidopsis*. For instance, ethylene-insensitive protein 2 (EIN2) is a vital component of the ethylene signalling pathway governing plant growth and development in *Arabidopsis* [49]. In our study, a novel intergenic lncRNA of *EIN2*, *MLAFT.18383.1*, exhibited elevated expression in floral buds and was positively regulated by m^6^A modification (Fig. 5D). Based on these findings, we proposed that *MLAFT.18383.1* potentially participated in *Arabidopsis* stage development through the ethylene signalling pathway by interacting with its neighbouring protein-coding gene *EIN2* under the regulation of m^6^A modification. Moreover, CLE45 is known to influence pollen tube growth in response to fluctuations in ambient temperature during the reproductive stage of *Arabidopsis* [50]. Here we observed a downregulation and reduced m^6^A level on *AT1G69587*, an antisense lncRNA of *CLE45 *(Fig. 5D). Consequently, *AT1G69587* was modulated by m^6^A modification and potentially contributed to *CLE45* expression as a natural antisense transcript, thereby influencing *Arabidopsis* stage development.

In terms of polyadenylation, poly(A) tails of lncRNAs in 5-week floral buds tended to be slightly longer than those in 2-week seedlings (Fig. 5E). Nevertheless, no significant correlation was observed between the expression changes and poly(A) tail length differences for individual lncRNAs during stage development (Spearman’s *ρ* = -0.04, *p* > 0.05; Fig. 5F). This outcome suggested that the effects of poly(A) tail length on lncRNA expression were too intricate to be explained by a simple linear regression model, and additional information are imperative to correlate the polyadenylation to lncRNA expression.

## Discussion

The importance of lncRNAs in stage development in plants is increasingly appreciated. Recent advancements in RNA-seq technologies have revolutionized lncRNA research. Nanopore DRS stood out as it captures ultralong even full-length native RNA molecules, enables accurate delineation of transcript boundaries, and benefits a more comprehensive transcriptome assembly. Unlike the polymerase chain reaction amplification step in Illumina NGS, which introduces bias and artifacts, nanopore DRS directly sequences native RNA molecules and thus serves as a precise method for RNA identification and quantification. Nevertheless, the limitations of nanopore DRS in terms of throughput and coverage have hindered the detection of rare or low-abundance transcripts. To address this, here we leveraged our recently developed pipeline tailored for nanopore DRS transcriptome assembly, known as LAFITE, to capture novel lncRNAs in *Arabidopsis* [27]. By exploiting the superiority of LAFITE to detect low-abundance transcripts, we identified 1149 novel lncRNAs utilizing an extensive collection of nanopore DRS datasets from wild-type *Arabidopsis*. This finding expanded the current Araport11 annotation of *Arabidopsis*, laying a foundation for investigating the landscapes of m^6^A modification and polyadenylation on lncRNAs.

Nanopore DRS also allowed us to directly capture native m^6^A modification sites from raw electrical signal fluctuations. By depicting the m^6^A landscape on lncRNAs, we unveiled the preferential location of m^6^A-modified sites on the AAACH 5-mer motif and the positive contribution of m^6^A modification to lncRNA expression. Moreover, we observed a positive correlation between expression changes and m^6^A modification variances during stage development, indicating a potential regulatory role of lncRNAs in *Arabidopsis* development. Here we called m^6^A using m6Anet, a tool developed on a multiple instance learning framework. To train a model for m^6^A detection in *Arabidopsis*, m6Anet obtained methylated sites by comparing the DRS data with low methylation from *vir-1* knockout plants to a control from *vir-1* complemented plants. Nevertheless, additional components such as MTA, MTB, FIP37 and HAKAI, and methyltransferases like FIO1 and ATMETTL5, have been reported to be responsible for installing m^6^A modifications in *Arabidopsis* [6–13]. Hence, the knockout of *vir-1* alone might not have completely eliminated m^6^A modifications on RNAs, potentially introducing intrinsic bias into the training model and m^6^A modification detection in *Arabidopsis*. To mitigate this issue, an alternative strategy involving in vitro transcribed (IVT) RNA libraries has been proposed [51]. However, further comparisons are necessary to evaluate the accuracies of different strategies for m^6^A detection. In light of these considerations, m6Anet was a reasonable choice for our current study, given its exceptional performance to date [52].

Although m^6^A modifications on lncRNAs are prevalent, the specific mechanism of m^6^A deposition on lncRNAs in *Arabidopsis* remains uncharacterized. In this study, we recruited diverse nanopore DRS datasets from mutants of MTA complex subunits and FIO1 to investigate the potential m^6^A modification mechanisms on lncRNAs. The preliminary findings revealed the complexity of the m^6^A modification mechanisms involving these subunits or methyltransferases, providing implications for subsequent mechanism investigations. Firstly, a multitude of methylated sites were detected in wild-type and all types of mutant samples, indicating the existence of potential mechanisms apart from the known subunits and methyltransferases that govern m^6^A modifications on these positions. Notably, the mutation of VIR appeared to introduce a considerable amount of novel m^6^A-modified sites, hinting at potential crosstalk among VIR-mediated m^6^A modifications. Finally, it should be noted that additional information and further experimental validation are imperative to confirm these implications.

The ability of nanopore DRS to directly capture full-length RNA molecules enabled us to precisely estimate poly(A) tail lengths. By characterizing poly(A) tail lengths on lncRNAs, we revealed that poly(A) tails assisted in lncRNA abundance by stabilizing the transcripts and protecting them from degradation. However, the stabilization and protection from poly(A) tails were not dependent on their lengths, indicating the intricate roles of polyadenylation in lncRNA abundance. Consequently, further interpretation and validation of the correlation between polyadenylation event and lncRNA expression are necessary.

In addition, we delved into investigating the correlations between m^6^A characteristics and poly(A) features on lncRNAs. At the transcript level, we found that the poly(A) tail length did not exhibit significant differences between methylated and unmethylated lncRNAs. Spearman’s rank correlation analysis revealed no significant correlation between poly(A) tail length and the modification ratio of individual lncRNAs. Furthermore, we explored the potential impact of the m^6^A sites closest to the TTS within 1000 nt to the poly(A) tails. Despite this proximity, we did not observe significant variations in poly(A) tail lengths between lncRNAs with or without m^6^A modification. Moreover, there was no significant Spearman’s rank correlation between the proximal m^6^A sites and poly(A) tail lengths. In conclusion, while polyadenylation event has been associated with m^6^A modification on the 3’ UTR of protein-coding RNAs, further investigations are necessary to elucidate the correlation between poly(A) tail length and m^6^A modification on lncRNAs [53].

In our investigation of lncRNA expression dynamics during *Arabidopsis* stage development, we gained valuable insights into the developmental regulation of lncRNAs in this model plant. We observed stage-specific and differential expression patterns of lncRNAs, suggesting their potential roles in specific tissues or stages, including developmental regulation. By integrating the information on m^6^A modifications and polyadenylation events, we explored the potential regulatory roles of these features in modulating lncRNA expression dynamics and their downstream functional roles. For instance, the novel intergenic lncRNA *MLAFT.18383.1* was positively regulated by m^6^A modifications on it, exhibited elevated expression in the reproductive stage, and potentially participated in the ethylene signalling pathway by interacting with its neighbouring protein-coding gene *EIN2*. On the other hand, the annotated antisense lncRNA *AT1G69587* was potentially involved in pollen tube growth by modulating *CLE45* expression under m^6^A modification regulations in the reproductive stage of *Arabidopsis*. Our findings shed light on the complex roles of epigenetic modifications and post-transcriptional processing in regulating lncRNA expressions and their functions during *Arabidopsis* development.

In the future, it would be valuable to delve into additional m^6^A methyltransferases, explore the crosstalk among multiple m^6^A sites on the same lncRNAs, and investigate the interplay among various epigenetic modifications to comprehend their combined influence on lncRNA expressions and functions. These pursuits hold the promise of revealing additional layers of complexity within the regulatory networks involved in plant developmental processes.

## Conclusions

In this study, we expanded our knowledge of the repertoire of lncRNAs in *Arabidopsis* and comprehensively delineated the m^6^A and polyadenylation landscapes on lncRNAs using nanopore DRS data. Additionally, we delved into the potential regulatory roles of this epigenetic modification and post-transcriptional processing on lncRNA abundance during stage development, spanning from the vegetative stage to the reproductive stage. Our findings provided valuable insights into the field of lncRNA research, particularly in terms of post-transcriptional regulation, and paved the avenue for further functional investigations of lncRNAs.

## Electronic supplementary material

Below is the link to the electronic supplementary material.

Supplementary Material 1

Supplementary Material 2

Supplementary Material 3

Supplementary Material 4

Supplementary Material 5

Supplementary Material 6

## Data Availability

The nanopore DRS data are available in the Sequence Read Archive (SRA) under the accession number PRJNA605023, and the European Nucleotide Archive (ENA) under the accession numbers PRJEB32782, PRJEB45935 and PRJNA749003. The Illumina NGS data are accessible in the SRA under the accession number PRJNA525820.

## Abbreviations

CDS: Coding sequence
CPM: Counts per million
DRS: Direct RNA sequencing
FIO1: FIONA1
FIP37: FKBP12-interacting protein 37 kDa
LAFITE: Low-abundance Aware Full-length Isoform clusTEr
LncRNA: Long non-coding RNA
m^6^A: *N*^6^-methyladenosine
MTA: mRNA adenosine methylase
NGS: Next-generation sequencing
Poly(A): Polyadenine
TG-ROC: Two-graph receiver operating characteristic
TPM: Transcripts per million
TSS: Transcription start site
TTS: Transcription termination site
UTR: Untranslated region
VIR: VIRILIZER
VIRC: *Vir-1* complemented
