# Supplementary material for: Nanopore direct RNA sequencing reveals N6-methyladenosine and polyadenylation landscapes on long non-coding RNAs in Arabidopsis thaliana
Source: BMC Plant Biol. 2024 Nov 26;24:1126. doi: 10.1186/s12870-024-05845-4 (PMC11590578; doi:10.1186/s12870-024-05845-4)
Supplement: Supplementary file 1 — Supplementary Material 1 [file 12870_2024_5845_MOESM1_ESM.docx]

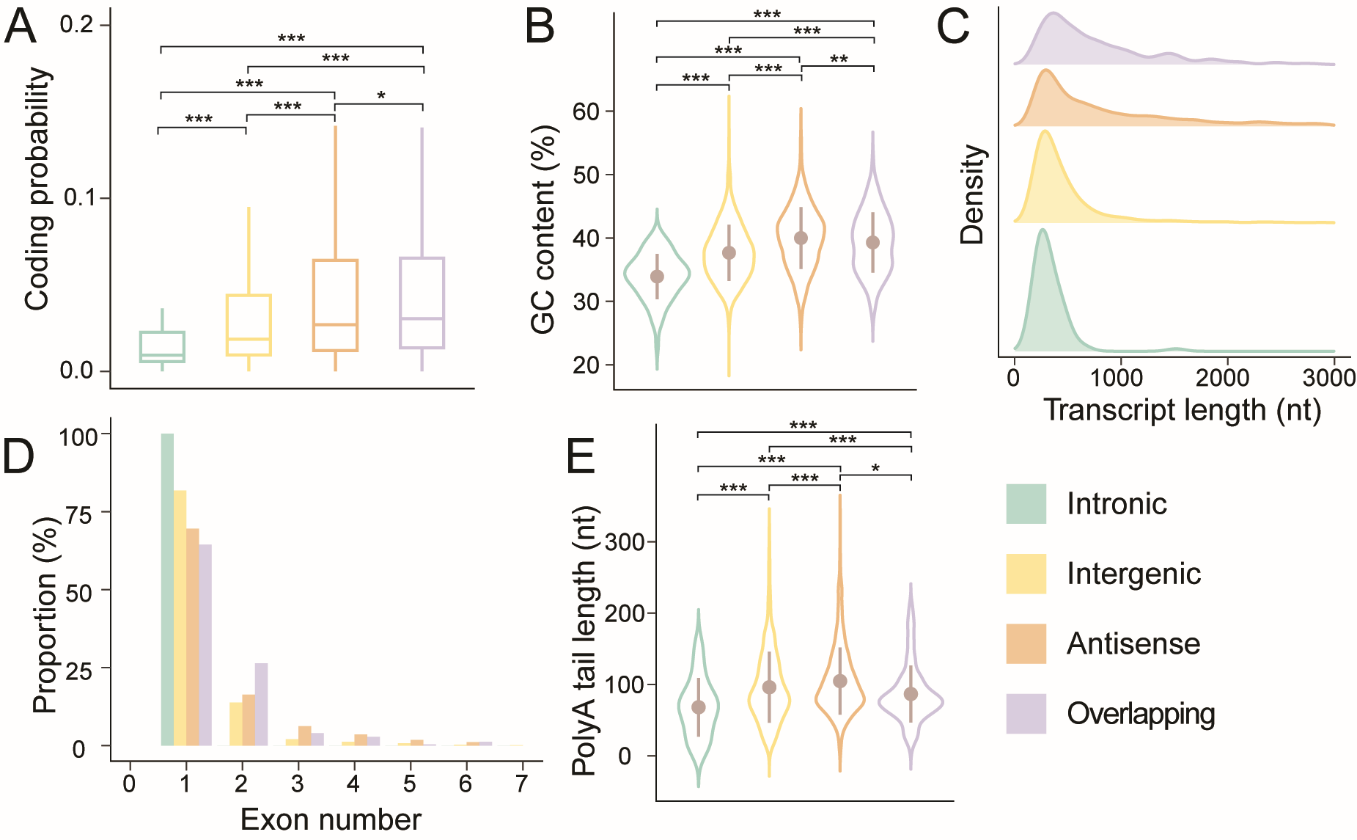


**Fig. S1** Characterization of lncRNAs in different categories. **(A)** Box plot comparing the coding probabilities among intronic lncRNAs (green), intergenic lncRNAs (yellow), antisense lncRNAs (orange) and overlapping lncRNAs (purple) (Mann-Whitney *U* test; ***, *p* < 0.001; *, *p* < 0.1). **(B)** Violin plot comparing the GC contents among intronic lncRNAs (green), intergenic lncRNAs (yellow), antisense lncRNAs (orange) and overlapping lncRNAs (purple) (Mann-Whitney *U* test; ***, *p* < 0.001; **, *p* < 0.05). **(C)** Density plot presenting the distributions of transcript lengths of intronic lncRNAs (green), intergenic lncRNAs (yellow), antisense lncRNAs (orange) and overlapping lncRNAs (purple). **(D)** Histogram showing the exon numbers of intronic lncRNAs (green), intergenic lncRNAs (yellow), antisense lncRNAs (orange) and overlapping lncRNAs (purple). (E) Violin plot comparing the poly(A) tail lengths among intronic lncRNAs (green), intergenic lncRNAs (yellow), antisense lncRNAs (orange) and overlapping lncRNAs (purple) (Mann-Whitney *U* test; ***, *p* < 0.001; *, *p* < 0.1).


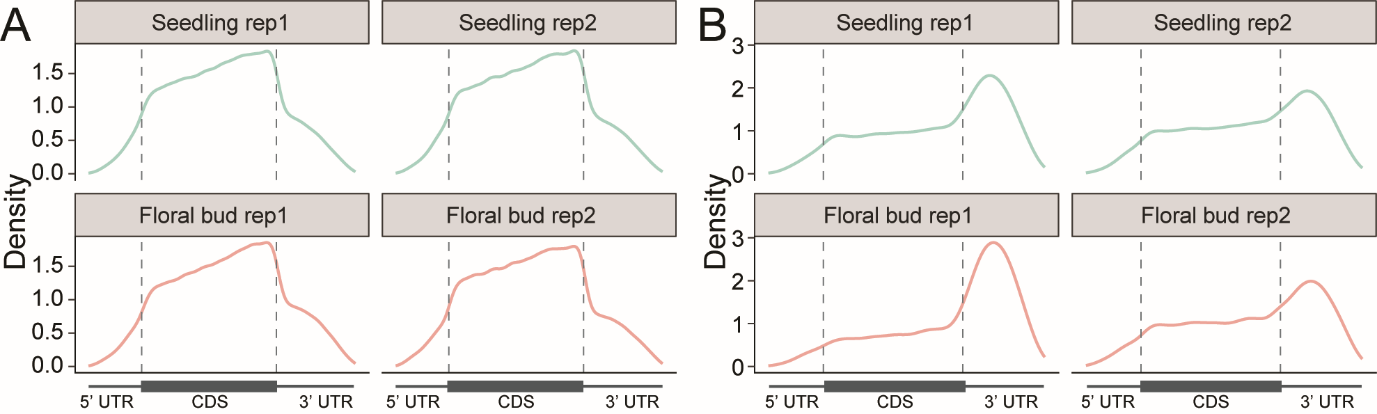


**Fig. S2** Metagene profiles showing the relative positions of **(A)** all m^6^A methylated sites and **(B)** highly m^6^A methylated sites with modification ratios exceeding 0.4 along different regions of protein-coding RNAs.

**
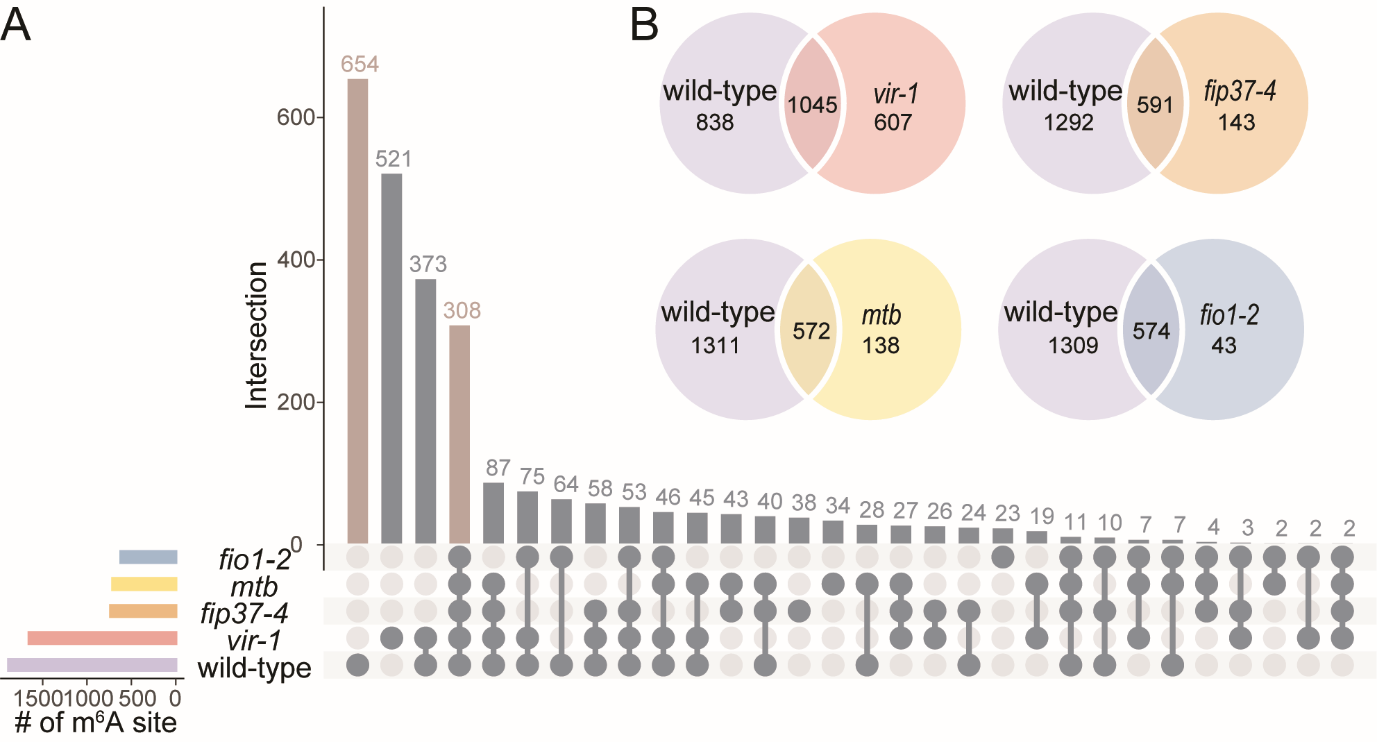
**

**Fig. S3** Numbers and intersections of m^6^A methylated sites detected in wild-type samples and various types of mutant samples.
